# Galactose as novel target against *Acanthamoeba* cysts

**DOI:** 10.1371/journal.pntd.0007385

**Published:** 2019-07-26

**Authors:** Ayaz Anwar, Naveed A. Khan, Ruqaiyyah Siddiqui

**Affiliations:** Department of Biological Sciences, School of Science and Technology, Sunway University, Subang Jaya, Selangor, Malaysia; National University of Singapore, SINGAPORE

## Introduction

*Acanthamoeba* spp. are the common free-living amoebae known to be associated with brain infections and sight-intimidating keratitis [1]. *Acanthamoeba* spp. have two stages in their life cycle: metabolically active trophozoite and dormant cyst [2]. A major challenge in the treatment of *Acanthamoeba* infections is degradation of resistant cysts. The process of conversion of trophozoite to cyst is called encystation, which is usually triggered by harsh conditions such as nutrition deprivation, extremes in environment, chemotherapy, etc. Once the treatment is stopped, cysts convert back into the infective trophozoites stage, resulting in infection recurrence [3]. It is imperative to target the cyst stage for successful prognosis. During encystation, the trophozoites efflux excessive food and enclose themselves in minimal metabolic activity by formation of double-walled (ectocyst and endocyst) protected cysts. The chemical composition of cyst walls of *Acanthamoeba* belonging to the T4 genotype consists of 35% carbohydrates and 33% proteins, and the rest is a mixture of lipids, ash, and some unidentified materials. Among the carbohydrates, galactose and glucose monosaccharides are present in combined 96%, while 3-linked galactopyranose is the most abundant glycosyl linkage in cyst walls, making up to 29% (Table 1) [4]. Hence, carbohydrates affecting agents are potential drug targets against cysts. In this regard, galactose has not gained deserved attention as a therapeutic target as compared to glucose. The product of glucose in the cyst walls has been identified as cellulose, and an extensive cellulose-targeting agent has been tested and found effective in either controlling encystation and/or a development of combination therapy [5–7]. Recently, Garajová and colleagues studied the effects of cytoskeletal elements such as microtubular networks and actin filaments in the last stages of encystment of *Acanthamoeba*. Cellulose fibrils, which are the main component of endocyst, were demonstrated in endocystic space as well as in the ectocyst. They further detected the intramembranous particles (IMPs) to form clusters in the cytoplasmic membrane during encystment by using a variety of microscopic techniques. Cyst wall impermeability due to the presence of complex polysaccharides has been shown to be responsible for the resistance of *Acanthamoeba* cysts against therapy [8]. In another report, Lloyd reviewed the aerophilic biochemical processes involved in the ecystment of *Acanthamoeba*, while also highlighting the effects of the most commonly used drugs, natural compounds, and contact lens solutions against cysts [9]. However, the most abundant carbohydrate, i.e., galactose, has been neglected. In this contribution, we highlight the importance of this untapped research area for specific drug development by identifying potential products of galactose.

**Table 1:** Comparative carbohydrates composition in the cyst walls of *Acanthamoeba castellanii*.

Monosaccharide	%	Glycoside linkage	%
Glucose	53.4	4-linked glucopyranose	22.2
Galactose	43.7	3-linked galactopyranose	28.6

## Possible products of galactose in *Acanthamoeba* cysts

**Fig 1:**
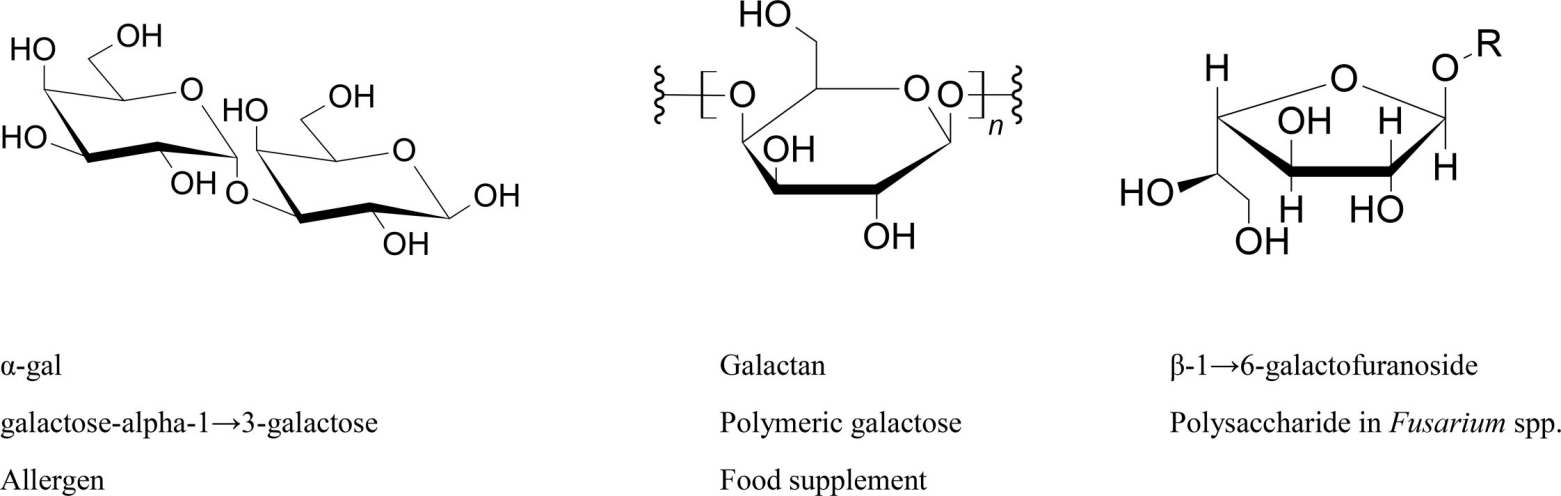
Galactose products most probably present in cyst walls of *Acanthamoeba castellanii*.

Galactose is synthesized by the human body, in which it forms part of glycolipids and glycoproteins in several tissues. It is also an important component of cerebrosides, which are glycosphingolipids that are important components in animal muscle and nerve cell membranes. Myelin is the most known cerebroside. Galactocerebrosides are typically found in neural tissue, while glucocerebrosides are found in other tissues. Moreover, in the human body, glucose is changed into galactose in order to enable the mammary glands to secrete lactose. One of the known products of galactose in *Acanthamoeba* cyst is galactose-alpha-1→3-galactose (alpha-gal) [4]. It is a disease-causing sugar that has been recognized as a potential allergen present in mammal meat. Alpha-gal is hydrolyzed into two units of galactose by alpha-galactosidase; however, unlike chemical hydrolysis of most of the sugars by using acid/base reactions, it is an unfeasible option for biological system. Another known product is polymeric galactose that is known as galactan, which is found in hemicellulose. Lipophosphonoglycan analysis of *Acanthamoeba* has shown the presence of galactose in the glycans both in the neutral form as well as in the amino form [10]. The cellulosome structure of various bacteria including *Clostridium thermocellum*, *Roseovarius albus*, etc., which are reviewed by Lakhundi and colleagues [11], has been studied via a combination of various biochemical, structural, and genetic analysis. It is also important to note that cellulosomal components of these microbes do not only include cellulases but hemicellulases as well. Galactans can also be degraded by hemicellulases. These are typically specific toward a particular sugar; therefore, we can find β-D-galactanases, β-D-mannanases, and β-D-oxylanases. Unlike cellulose, hemicellulose is easier to degrade, but strangely, none of the hemicellulases have been tested against amoebae, while cellulase has shown potential in combination therapy against *A*. *castellanii* [5]. In a recent report, *Bacillus subtilis* is shown to possess different enzymes for the utilization of plant cell wall polysaccharides. This galactan degradation cascade includes a gene cluster containing galactan degradation genes (*ganA* and *ganB*), two transporter component genes (*ganQ* and *ganP*), and the sugar-binding lipoprotein-encoding gene *ganS* [12]. Another product of naturally occurring galactose was identified by chemical structural analysis of the carbohydrate chains in the glycoproteins of fungi *Fusarium* spp. M7-1 demonstrated that the backbone of the polysaccharide chain is beta-1→6-galactofuranoside linkages [13]. However, the presence of these suggested galactose products in *Acanthamoeba* as well as in other parasites are all not biochemically proven to be present, which needs to be addressed urgently. The abundance of galactose found in cyst walls suggest the presence of various compounds, which might be completely unknown as well. Therefore, the galactose component of *Acanthamoeba* cysts should be subjected to thorough spectroscopic and chromatographic identification so that further understanding of the possible pathways could be developed, which will lead to novel drug targets. Some of the possible biologically known products of galactose are presented in Fig 1 below. Based on the highlighted product, the next section deals with some of the important galactose targeting strategies that can be utilized to target *Acanthamoeba* cysts.

## Glycoside-targeting biomolecules

Alpha-galactosidase hydrolyses the terminal alpha-galactosyl sugars from glycolipids and glycoproteins and may be a useful enzyme to target biosynthesis and/or degradation of galactose in *Acanthamoeba*, which may result in compromised cyst walls integrity. Another enzyme lactase may be useful against *Acanthamoeba* cysts, as this is essential to metabolize the lactose sugar into glucose and galactose, primary subunits in cyst walls of *Acanthamoeba*. Previously, biomolecules including cellulase, glycogen phosphorylase–signaling RNA, etc. are known to alter formation, degradation, and viability of *Acanthamoeba* cysts; however, these biomolecules are restricted to target glucose [11,14]. Endo-β-galactofuranosidase has been used to degrade beta-1→6 galactofuranoside linkages present in the fungi of *Fusarium* spp. [11]. These biomolecules can be potential candidates for targeting galactose in the cyst walls of *Acanthamoeba* species. Furthermore, the knowledge extracted from allergic reactions involving metabolism of galactose can be applied to target *Acanthamoeba* cysts. Antihistamine drugs such as diphenhydramine and epinephrine are usually given to control the reaction of alpha-gal allergy. Small molecule activators of glycoside hydrolases are another interesting candidate that may be effective with or without the enzyme to accelerate the catalysis of the targeting sugar [15]. Furthermore, formulating nanoparticles conjugated with the above mentioned molecules to specifically target galactose may exhibit improved cysticidal effects against *Acanthamoeba*. Nanoparticles are reported to deliver drugs effectively at the target site which enhance drug efficacy [16]. If proven successful, the proposed strategy would offer a viable option for broadening the molecular targeting of *Acanthamoeba* cysts and a major step forward in tackling the drug resistance in *Acanthamoeba* species.

## Conclusion

We anticipate that galactose-targeting approaches will open new horizons in the drug development against *Acanthamoeba* cysts. Although, some common products of galactose are identified here that are known to be present in biological systems of parasites; however, there can be a lot more different compounds that are not known yet and need further identification. Hence, isolation and structural analysis of complex glycosides present in cyst walls by spectroscopic techniques guarantees further understanding of resistant *Acanthamoeba* cysts. Furthermore, galactose-targeting molecules and nanomaterials suggested here can provide a novel therapeutic target against *Acanthamoeba* cysts.
